# Baroreflex Activation Therapy Invasive Hemodynamic “Reverse Ramp” Titration

**DOI:** 10.1016/j.jscai.2026.104400

**Published:** 2026-03-24

**Authors:** Frazer A. Tessema, Jonathan Grinstein, Daniel Burkhoff, Mark N. Belkin

**Affiliations:** a Section of Cardiology, The University of Chicago Medicine, Chicago, Illinois; b Cardiovascular Research Foundation, New York, New York

**Keywords:** baroreflex activation therapy, heart failure with reduced ejection fraction, hemodynamics

Baroreflex activation therapy (BAT) is a technology for heart failure with reduced ejection fraction (HFrEF) that applies direct current to carotid baroreceptors to promote vagal activation.^1^ Patients with HFrEF typically have aberrant carotid body function secondary to heart failure and other potentially comorbid conditions such as hypertension.^1^ The underlying effect of BAT on the autonomic nervous system is to restore sympathovagal balance, which results in systemic physiologic changes, such as reducing filling pressures and increasing venous capacitance, that promote improvement in heart failure symptoms.^2^

BAT has previously demonstrated efficacy in treating symptomatic expression of HFrEF, specifically improving functional status, quality of life, and exercise capacity.^1^ In experimental studies and case series, BAT has improved left ventricular function and partially reversed left ventricular remodeling at both the molecular and cellular levels.^1^^,^^2^ BAT holds particular promise to promote hemodynamic support in patients with HFrEF on maximum guideline-directed heart failure therapy, an N-terminal pro-B-type natriuretic peptide less than 1600 pg/mL, and refractory symptoms. We describe acute hemodynamic changes associated with BAT dose titration.

A 71-year-old man with HFrEF on maximally tolerated guideline-directed therapy, including cardiac resynchronization therapy, underwent successful BAT implantation followed by dose titration to 7.2 mA.

**Figure 1:**
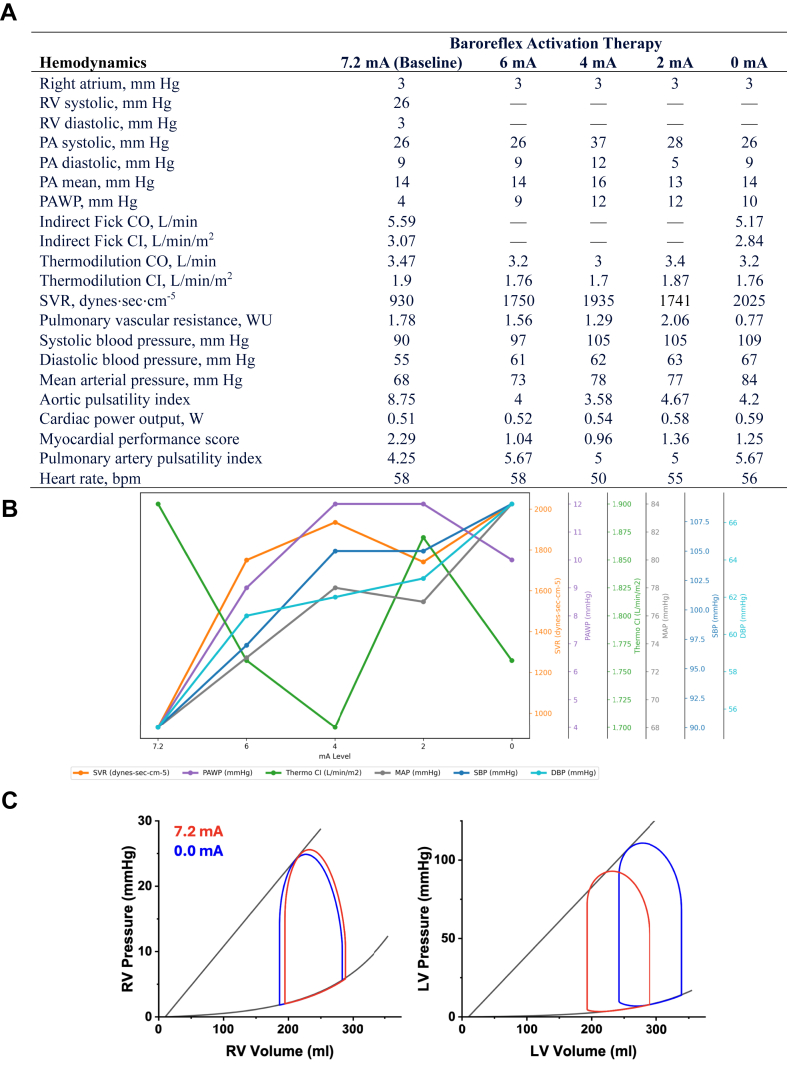
**Invasive hemodynamic measurements during baroreflex activation therapy (BAT) titration.** (A) Invasive hemodynamic measurements during BAT titration. Standard and advanced hemodynamic metrics are noted at each step of BAT titration. (B) Hemodynamic trends during BAT titration. Note increases in systemic vascular resistance, systemic blood pressure, and pulmonary artery wedge pressure, along with concomitant reduction in cardiac output, as measured by thermodilution. (C) Biventricular pressure-volume loop analysis created via validated modeling (HARVI). Note ventricular-arterial uncoupling with BAT down-titration. CI, cardiac index; CO, cardiac output; DBP, diastolic blood pressure; LV, left ventricle; MAP, mean arterial pressure; PA, pulmonary artery; PAWP, pulmonary artery wedge pressure; RA, right atrium; RV, right ventricle; SBP, systolic blood pressure; SVR, systemic vascular resistance.

Informed, written consent was obtained to record and measure hemodynamics prior to the procedure. The study was carried out in accordance with appropriate ethical guidelines. Right heart catheterization occurred via the right internal jugular vein. After obtaining baseline measurements, BAT was down-titrated from the baseline 7.2 mA to 6.0, 4.0, 2.0, and 0.0 mA (Figure 1A, B). Measurements were recorded after 2 minutes at each stage. As BAT output decreased from 7.2 to 0.0 mA, pulmonary artery wedge pressure increased (4 to 10 mm Hg) and cardiac index decreased (thermodilution 3.5 to 3.2 L/min/m^2^, indirect Fick 3.07 to 2.84 L/min/m^2^) as systemic vascular resistance increased (930 dynes⋅sec⋅cm^−5^ to 2025 dynes⋅sec⋅cm^−5^) (Figure 1A, B). The advanced hemodynamic metrics aortic pulsatility index and myocardial performance score decreased linearly along with BAT output.

In this hemodynamic case, BAT down-titration resulted in increasing afterload and preload along with decreasing contractility, as evidenced by the changes in systemic vascular resistance, filling pressures, and cardiac index. These hemodynamic changes represent ventriculo-arterial (VA) uncoupling, in which the ratio of afterload (E_a_) to contractility (E_es_) becomes less optimal, representing worsening cardiac inefficiency. The decreasing aortic pulsatility index and myocardial performance score noted in this case have been associated with reduced cardiac efficiency, power, and VA coupling.^3^^,^^4^ Similarly, pulmonary artery pulsatility index and right ventricular myocardial performance score decreased with BAT output reduction, a phenomenon correlated with right ventricle power and efficiency.^5^ These hemodynamic changes of VA uncoupling are illustrated through pressure-volume loop modeling (HARVI) (Figure 1C). These findings mirror a recent case linking BAT to improved VA coupling, as measured through a noninvasive, echocardiographic-based method rather than invasive hemodynamic assessment.^2^

The acute, invasive hemodynamic changes with down-titration of BAT observed in this case study indicate VA uncoupling, highlighting a potential mechanism of action of this therapy. Further invasive hemodynamic evaluation of BAT may help further elucidate its acute cardiovascular effects on biventricular function.

**Pearls in Hemodynamics**

• Baroreflex activation therapy (BAT) is used to treat chronic heart failure with reduced ejection fraction via stimulation of the carotid baroreceptors.
• This is the first published account of acute, invasive hemodynamic changes related to dose titration of BAT, which confirms the expected association with changes in measures of cardiac afterload and subsequent preload and contractility.
• The acute, invasive hemodynamic changes noted during BAT down-titration are representative of ventricular-arterial uncoupling, as illustrated through pressure-volume loops.
